# Mixed Oxides: Role of Washing and Residual Ions in Transesterification Reactions

**DOI:** 10.1021/acsomega.5c08243

**Published:** 2025-10-28

**Authors:** David Kocián, Martin Hájek, Karel Soukup, Luděk Kaluža, Rostislav Prokeš, Miroslava Bérešová, Jakub Vagunda

**Affiliations:** † 495954 University of Pardubice, Faculty of Chemical Technology, Studentská 573, 532 10 Pardubice, Czech Republic; ‡ Institute of Chemical Process Fundamentals of the CAS, Rozvojová 135, 165 00 Prague 6, Czech Republic; § 561603 VSB—Technical University of Ostrava, Centre for Energy and Environmental Technologies, ENET Centre 17. listopadu 2172/15, 708 00 Ostrava, Czech Republic; ∥ 61791 Slovak University of Technology, Faculty of Chemical and Food Technology, Radlinského 9, 812 37 Bratislava, Slovak Republic

## Abstract

The mixed oxides (MOs) serve as catalysts for many reactions such as transesterification, transformation of ethanol to butanol, catalytic cracking, or dehydrogenation reactions. MOs are usually synthesized from hydrotalcites, which are often prepared by the coprecipitation method. However, some chemicals can remain after coprecipitation and influence the properties of MOs, including subsequent applications. The novelty lies in investigating how the residual chemicals affect the properties of hydrotalcites, MOs, and the transesterification reaction (conducted in both one- and two-step processes). Mg–Al and Mg–Fe hydrotalcites were synthesized from chloride and nitrate salts via coprecipitation with NaOH, followed by washing with varying amounts of redistilled water, resulting in variations in the sodium ion content (more water, less sodium ions). All materials were characterized by many analytical methods such as X-ray diffraction, metal determination, scanning electron microscopy, textural properties, and basicity determination. MOs synthesized from chlorides contained stable NaCl, which is not catalytically active, and blocked the pores, leading to a reduced surface area and, consequently, a lower transesterification yield. In contrast, MOs prepared from nitrates contained unstable NaNO_3_, which decomposed during calcination and, upon exposure to water, formed basic species (NaOH) that promoted transesterification. Therefore, the effect of residual sodium varies depending on the material precursors. This understanding helps us to improve the synthesis of hydrotalcites and mixed oxides.

## Graphical abstract

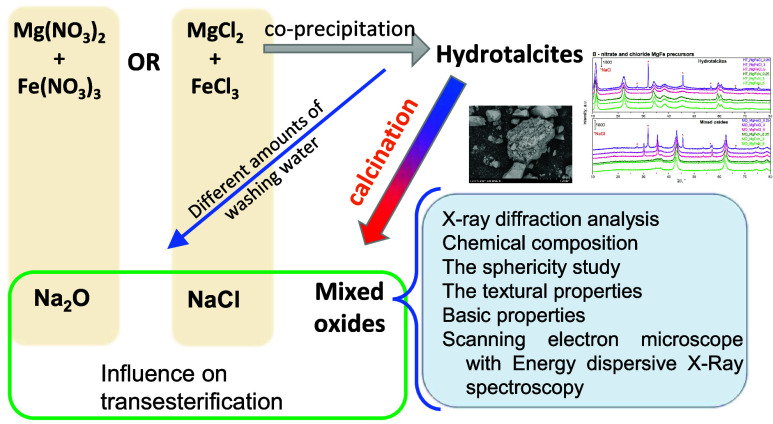

## Introduction

The layered double hydroxides (hydrotalcites or HTs) are an important class of materials and can be found in nature as materials with the general formula [M_1–*x*_^II^ M_*x*_^III^(OH)_2_]­[X_*x*/*q*_^*q*–^·*n*H_2_O], where M^II^ and M^III^ represent di- and trivalence metals, respectively; *x* is the molar fraction of M^III^; and *q* is the charge of an anion. The structure of hydrotalcites consists of layers of di- and/or trivalence metal cations (such as Mg^2+^, Ni^2+^, Ca^2+^, Al^3+^, Fe^3+^, etc.), between which anions (such as CO_3_^2–^, OH^–^, SO_4_^2–^, Cl^–^, etc.) and water molecules are located. The HT can be used (i) as a drug carrier due to their layered structure, which allows the controlled release of drugs, (ii) for the production of nanocomposites, and (iii) for removal of pollutants from water and air due to their ability to absorb various ions and molecules. The mixed oxides are synthesized through the calcination of HTs (heating hydrotalcites to 400–500 °C) and are used as catalysts for various reactions such as transesterification,^1^ hydrogenolysis of glycerol,^2^ production of higher alcohols,^3^ CO_2_ capture, and other organic syntheses.^4^

Transesterification, as a typical acid–base reaction, was selected as a model reaction for an acid–base catalyst (mixed oxides). It is a reaction between triglycerides (main components of vegetable oils) and a low-molecular-weight alcohol (usually methanol) to form a mixture of fatty acid methyl esters and glycerol. The esters have several other applications, such as biofuels, solvents, lubricants,^5^ or pharmaceutical products,^6^ whereas glycerol is used as additives to food,^7^ beauty products, or as precursors for the synthesis of drugs, polyether, or alkyd resins.^8^ A homogeneous catalyst (typically KOH or NaOH) is used industrially due to its low reaction temperature (60 °C), shorter reaction time than heterogeneous catalysts, and lower required amounts of catalyst. However, drawbacks are especially the catalyst’s nonreusability and soap formation, which contaminates glycerol.

The hydrotalcites can be synthesized by four methods: (i) coprecipitation (the most often used), (ii) hydrolysis, (iii) sol–gel,^9^ or (iv) microwave.^10^ Coprecipitation is usually carried out from nitrates of metals at a constant pH, which is maintained by addition of basic solutions, such as KOH, NaOH, or Na_2_CO_3_. However, when hydrotalcites are washed and filtered (from the slurry after synthesis) by redistilled water, some contaminants can remain in their structure. The chemical residues influence the properties of hydrotalcites and mixed oxides as well as their applications. In recent years, various mixed oxides (Mg–Al,^11^ Ca–Zn,^12^ Mg–Ti–Zr, or Mg–Fe^13^) were studied to address the issues of residue compounds after the synthesis and their influence on transesterification, carboxylation of crude glycerol,^14^ or valorization of alcohols.^15^ The significant influence of residual ions on transesterification was found, but the materials were not characterized in detail.^16^ Fraile et al. published transesterification of methyl palmitate with isobutanol catalyzed MO with various metals (Mg–Al, Mg–La, and Mg–Ga): the biodiesel yield increased with increasing residual sodium amounts, but only X-ray diffraction (XRD) was used for characterization.^17^

Mg–Al mixed oxides were often synthesized only from nitrates but without comparison with different anions (such as chlorides) or with different metals in mixed oxides (such as iron), which is a novelty in this paper. Furthermore, various methods were used in this study such as chemical composition, structure, basic properties, adsorption isotherms, sphericity, etc. Detailed characterization will provide insight into the influence of sodium compounds on the material properties, and, consequently, on the transesterification of vegetable oil.

## Materials and Methods

The properties of HTs and mixed oxides (MOs) were studied by using various analytical methods. The properties of products of transesterification were compared using gas chromatography with a flame ionization detector (GC-FID).

### Preparation of Hydrotalcites and Mixed Oxides

The HTs were prepared by the coprecipitation method according to the study,^18^ with the molar ratios of Mg/Al and Mg/Fe of 3.5:1 (details are listed in Table 1). First, the chemicals (nitrates or chlorides of metals) were introduced into a 3 L glass reactor with a shaft stirrer (Heidolph, Germany) and stirred at 250 rpm. The automatic titrator (736 GP Titrino, Metrohm, Switzerland) filled with 2 mol/L NaOH solution was used to set the pH of the coprecipitated mixture to 10.0 ± 0.1. The mixture was left at 60 °C for 16 h under constant stirring for the crystalline phase to develop. Afterward, the mixture was filtered using a filter press (Školník Hobra, Czechia), and various amounts of water (0.25, 3, and 5 dm^3^) were used for washing the HT to obtain different amounts of sodium ions left in materials. Then, the materials were left on a Petri dish for 2 days to dry out.

**Table 1:** Amount of Precursors Used for Hydrotalcite Synthesis

HT sample	MgCl_2_·6H_2_O, g	AlCl_3_, g	FeCl_3_, g	Mg(NO_3_)_2_·6H_2_O, g	Fe(NO_3_)_3_·9H_2_O, g
HT_MgAlCl	70.0	12.9			
HT_MgFeCl	70.0		16.0		
HT_MgFeN				70.0	31.0

The MOs were prepared from HTs by calcination (4 h, 450 °C, and a speed of heating of 5 °C/min), which had been milled and sifted to obtain particles in the range 250–500 μm.

### Analytical Methods

The chemical composition of HTs and MOs was determined using inductively coupled plasma with mass spectrometry (7900 ICP-MS, Agilent Technologies, United States) with an inner Rh standard. The materials were dissolved at 50 °C in 4 mL of HNO_3_ in polytetrafluoroethylene cartridges (Speedwave XPERT device, Berghof Company, Germany). Each analysis was carried out three times and the average value is presented.

The XRD with JCPDS sheets, PDF 2-2002 was used to verify the successful formation of HTs and MOs after the synthesis and to study impurities in their structure. The powdered sample was presented into a measuring cell (MiniFlex 600, Rigaku, United States). The measurements were carried out from 2θ = 20 to 80° with the speed of rotation of 6°/min.

Scanning electron microscopy (SEM) with energy-dispersive X-ray (EDX) spectroscopy (TM4000Plus II, Hitachi, Japan) with an electron beam acceleration of 15 kV in deep vacuum was employed to study the surface of the catalysts. The chemical composition on the surfaces of the materials was determined in three different spatial positions for each sample.

The particle shape was determined by the Cilas 1190 particle size analyzer equipped with an integrated image analysis system that provides comprehensive particle characterization using Cilas ExpertShape V 4.14 software (Anton Paar, Les Ulis, France), which is a laser particle analyzer that measures particle size and particle shape.^19^ The method is crucial because the particle shape significantly affects its physical properties and behavior. Sphericity is defined as the ratio of the radius of the inscribed circle to the radius of the circumscribed circle, where the radius of the inscribed circle is the smallest distance between the outline and the particle’s center of gravity and the radius of the circumscribed circle is the largest distance between the outline and the particle’s center of gravity.

The temperature-programmed desorption of CO_2_ (TPD-CO_2_) was used to study the basicity of MOs. The measurement was performed on an AutoChem II 2920 (Micromeritics, United States) device equipped with a mass spectrometer (OmniStarTM GSD 320, Pfeiffer Vacuum, Germany). A detailed description can be found in the paper.^18^ The amount of desorbed CO_2_ was calculated from a calibration. The results were normalized, i.e., the signal was divided by its maximal value and plotted. The accuracy of the method is approximately ±5 rel %.

The specific surface of MOs was determined by N_2_ physisorption at 77 K on ASAP2020 and 2050 analyzers (Micrometrics, United States). The MOs were activated under vacuum at 350 °C for 12 h before the analysis. The specific surface area was calculated using the BET equation, and pore diameters were determined using the BJH model. The micropore content was determined by the *t*-plot (the Harkins–Jura master isotherm was used).

### Catalytic Activity

The catalytic activity of prepared MOs was tested in the transformation of vegetable oils to a mixture of esters (transesterification). The transesterification was carried out in two ways (one-step and two-step reactions), and their results were compared. The edible rapeseed oil was purchased from PREOL company (Lovosice, Czechia) with the following fatty acid profile: palmitic 4.7 wt %, stearic 1.9 wt %, oleic 60.5 wt %, linoleic 26.2 wt %, and linolenic 6.1 wt %. The density at 25 °C (0.962 g/cm^3^), kinematic viscosity at 40 °C (9.0 mm^2^/s), water content (410 ppm), acid number (0.15 mg KOH/g), peroxide value (2 mequiv/kg), and iodine value (107.1 g I_2_/100 g) of rapeseed oil were determined.

In the one-step transesterification, the mass of 1.0 g of the catalyst, 18.4 g of methanol (p.a., Lach:ner, Czechia), and 17.8 g of rapeseed oil (PREOL, Czechia) were introduced into a pressure reactor (4560 Mini Reactor, Parr, United States). The reaction conditions were set to 120 °C, 350 rpm, and 6 h. After the reaction, the mixture was cooled down to room temperature, and the catalyst was filtered off. Excess methanol in the mixture of products was distilled off by using an increased temperature and a decreased pressure (65 °C, 1 kPa), whereas the glycerol phase (if any was formed) was discarded. In the two-step transesterification, the mass of 80.0 g of rapeseed oil, 2.4 g of the catalyst (MO), and 55.2 g of methanol were introduced into a nickel–chromium steel batch pressure reactor (Parr Instruments, model 4520, United States). The reaction was carried out for 3 h at 140 °C with a stirring of 350 rpm according to the study.^20^ After the reaction, the mixture was cooled down to room temperature, the catalyst and methanol were removed, and glycerol (if it was present) was separated from the mixture of methyl esters and unreacted oil by centrifugation. The formed glycerol was removed, whereas the mixture of esters and oil was used again for transesterification with a new batch of catalysts under the same reaction conditions. The process of the purification of products was repeated.

The content of methyl esters in the ester phase was determined according to EN ISO 14103 using gas chromatography with a flame ionization detector (Shimadzu GC-2010, Japan) with an accuracy of ±2 rel %.

## Results and Discussion

The materials were signed according to (i) form (i.e., HTs for hydrotalcites and MOs for mixed oxides), (ii) metals in precursors (Mg, Al, or Fe), (iii) anions in precursors (Cl from chlorides or N from nitrates), and (iv) the amount of water used for washing HTs (0.25, 3, or 5 dm^3^). For example, HT_MgAlCl_0.25 is a hydrotalcite prepared from chlorides of Mg and Al and it was washed with 0.25 dm^3^ of water; MO_MgFeN_5 is a mixed oxide prepared from nitrates of Mg and Fe and it was washed with 5 dm^3^ of water prior to calcination.

### Characterization of Hydrotalcites and Mixed Oxides

Several analytical methods were applied to study the properties of the HTs and MOs. XRD was carried out to confirm the successful formation of HTs and MOs after the synthesis (Figure 1A,B).

**Figure 1:**
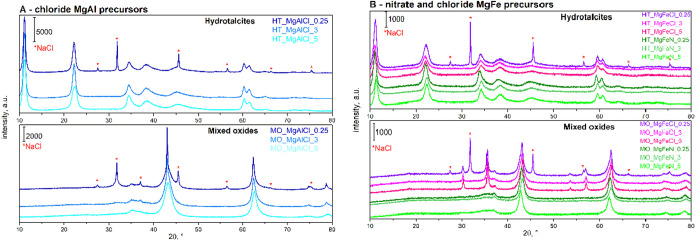
XRD diffractogram of MgAl (A) and MgFe (B) HTs and MOs.

The formation of HTs synthesized from magnesium and aluminum precursors was confirmed by the presence of diffraction lines 2θ ≈ 11.3, 22.6, 33.9, 38.2, 59.5, and 60.7°^21^ (Figure 1A) and similar lines were found for Mg–Fe hydrotalcites (Figure 1B). After calcination, the formation of mixed oxides was confirmed by the presence of diffraction lines 2θ ≈ 37.0, 43.1, 62.4, and 78.9° corresponding to the MgO phase^18,22^ and lines 2θ ≈ 30.1, 35.5, 53.5, 57.0, 62.4, and 74.1° corresponding to the FeO phase.^23^ Therefore, the successful synthesis of HTs and MOs was confirmed.

For chloride precursors (Figure 1A), the additional diffraction lines at 2θ ≈ 27.5, 31.8, 45.6, 56.6, 66.3, and 75.4° were detected for the least washed hydrotalcites (0.25 dm^3^ of redistilled water), which were identified as NaCl.^24,25^ NaCl was formed by the recombination of ions from NaOH (agent for pH regulation) and MgCl_2_/FeCl_3_/AlCl_3_ during the synthesis of HTs. The impurities of NaCl were also detected by XRD by the study of Chaillot et al., who synthesized HTs from chloride precursors under various conditions.^26^ On the contrary, no diffraction lines of NaCl were detected by XRD in other studies,^27,28^ where mixed oxides were prepared from chlorides (due to higher amounts of washing water). For nitrate precursors, no NaNO_3_ diffraction peaks were detected, unlike in our earlier HT synthesis^18^ likely because (i) the NaNO_3_ content was below the detection limit or (ii) the salt lacked sufficient crystallinity.

#### Chemical Composition

The real molar ratio of Mg/Fe and Mg/Al was determined by two different methods: ICP-MS (bulk analysis) and SEM-EDX (surface analysis). The molar ratio of Mg–Fe on the surface of the MO was between 2.2:1 and 2.6:1 for both forms (Cl and N); see the data in Table S1 (Supporting Information). In contrast, the molar ratio of Mg:Al was in the higher range of 3.5:1, 3.7:1, and 6.3:1 for MO_MgAlCl 5, 3, and 0.25, respectively. The molar ratios of Mg:Fe for chloride precursors in bulk were in the range of 3.2–3.4:1 and for nitrate precursors were in a lower range of 2.9–3.0:1, i.e., lower amounts of magnesium were present in materials from nitrate precursors. The reason is that magnesium in nitrates is more easily washed out from the structure of hydrotalcites than from chlorides (the solubility of MgNO_3_ is 71 g/100 mL, whereas that of MgCl_2_ is 55 g/100 mL in water, both at 25 °C^29^). These results indicate different chemical compositions on the surface and in the bulk of MOs.

In addition, the concentration of sodium was determined using both methods (Table 2). The amount of sodium in materials decreased with the increasing amount of washing water, which was expected and is in accordance with the previous study.^18^ According to SEM-EDX, the white flakes were observed in MO synthesized from chlorides and identified as NaCl because sodium and chlorine were in the same place (Figure 2 and Table S2 in the Supporting Information). NaCl was detected in all materials (heterogeneous distribution), which is consistent with the XRD results, where the diffraction lines for NaCl were also observed. These findings indicate that a fraction of NaCl remained in the structure after synthesis, which influences both the textural and catalytic properties of the materials.

**Figure 2:**
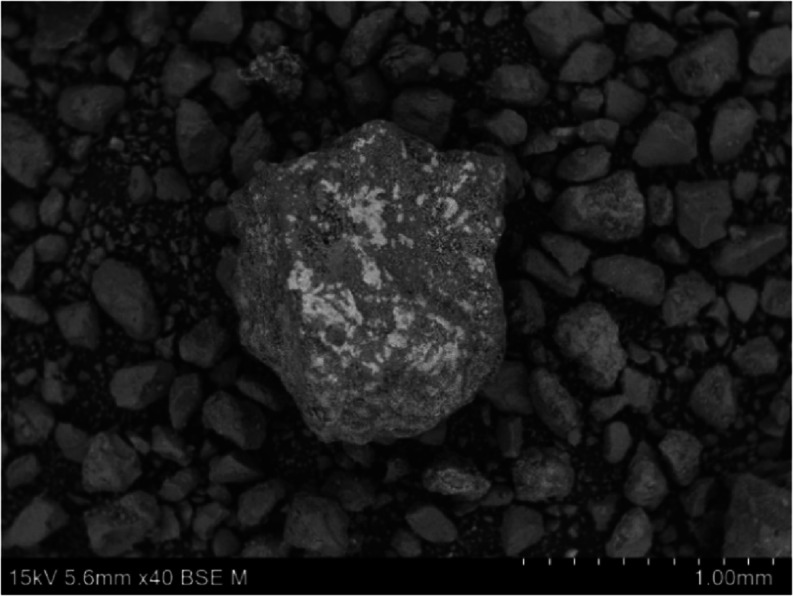
SEM-EDX image of MO_MgAlCl_0.25.

**Table 2:** Concentration of Sodium Ions in Mixed Oxides

			sodium content, mg/g	
HT	anion of precursors	water amount, dm^3^	ICP-MS	SEM-EDX
Mg–Al	Cl^–^	0.25	77.7	24.1
		3	8.4	3.9
		5	5.6	1.2
Mg–Fe	Cl^–^	0.25	47.8	52.5
		3	4.0	<1.0
		5	4.5	<1.0
	NO_3_^–^	0.25	5.8	6.1
		3	0.05	<1.0
		5	0.01	<1.0

No ‘flakes’ of sodium were detected in materials synthesized from nitrate precursors because sodium is more easily washed out from nitrates due to a higher solubility of NaNO_3_ in water (91.2/100 g at 25 °C) compared to NaCl (36.0/100 g at 25 °C).^30^

The sphericity study, which is rarely presented, is the novelty of this paper. The dependency of frequency on sphericity was determined for all mixed oxides (Figure S1). The sphericity in the range 0.45–0.65 (the sphericity one is of an ideal sphere) corresponded to SEM figures, where the irregular particles were found (Figure 2). The curves for all types of sets are similar; the MO with the lowest amount of washing water (0.25 dm^3^) had the narrowest distribution curve of sphericity, which broadened with increasing the amount of washing water.

The textural properties of mixed oxides, such as the type of isotherm, pore distribution, and specific surface area, were studied (Figure 3 and Table 3). Isotherms for all MOs were very similar (type IV), which is specific to mesoporous materials (Figure 3A). Furthermore, the hysteresis loop of type H1 was observed in all MOs, which is related to cylindrical pores, typical for this type of material.^31^ The most washed catalysts (with 5 dm^3^ of water) were chosen for the comparison of pore diameters because most of the impurities were removed by washing and different pore diameters were found (Figure 3B). The highest pore diameters were for MO_MgFe from nitrates (15–30 nm), medium for MO_MgFe from chlorides (9–11 nm), and the lowest for MO_MgAlCl (4–10 nm). Therefore, MOs from nitrates had higher pore diameters than those from chloride precursors. This agrees with a study from Hájek et al. who synthesized MgAl mixed oxides from nitrates and chlorides in a similar manner with average pore sizes of 40 and 25 nm, respectively.^32^ However, many authors published much smaller pore size diameters for various MOs from nitrate precursors such as Yun et al. for MgAl with an average pore size diameter of 2.5–2.8 nm.^33^ Nousir et al. observed pore diameters in the range 3.5–5.0 nm for CeZr.^34^ Bashah et al. synthesized Cr/Ca and Cr/Zn MO with pore diameters of 1.8 and 1.7 nm, respectively.^34^ Patra et al. synthesized TiO_2_–Fe_2_O_3_ MOs from iron chloride and titanium isopropoxide precursors with an average pore diameter of 3.1 nm.^35^ In conclusion, precursors, metals, and the ways of synthesis strongly influence the average pore size distribution.

**Figure 3:**
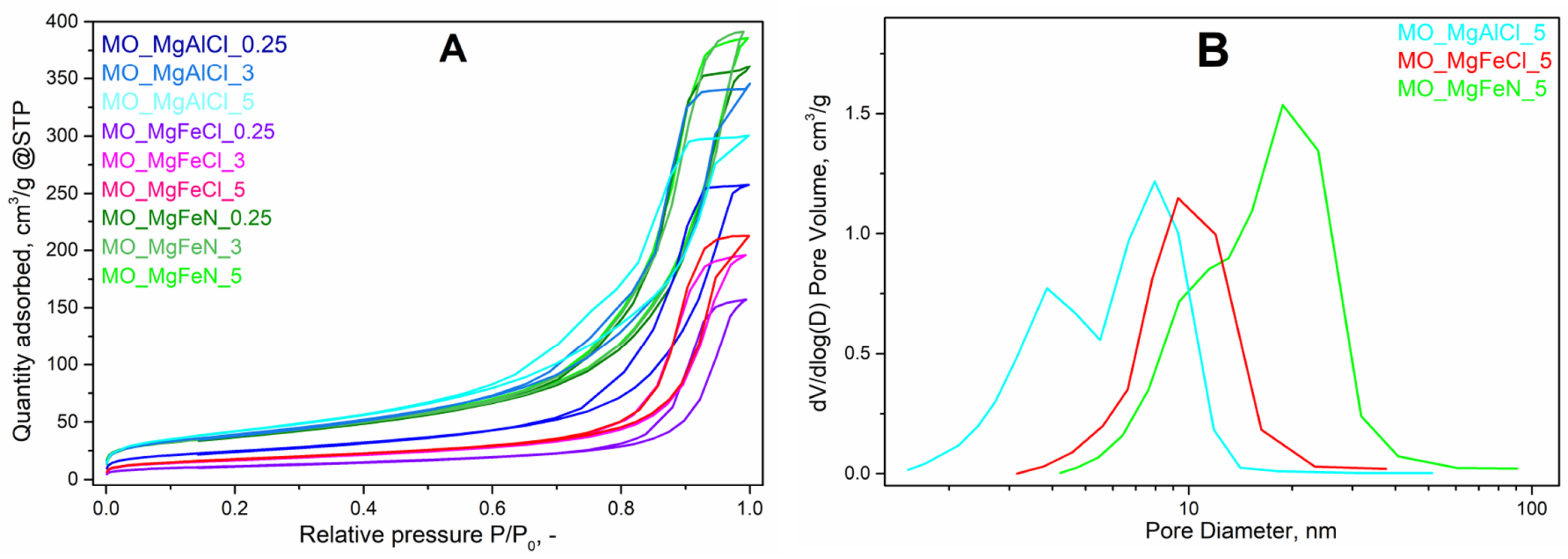
Adsorption isotherms (A) and distribution of pores (B) of mixed oxides.

**Table 3:** Textural and Basic Properties of Mixed Oxides and Ester Content of One-Step Transesterification^a^ *S*_BET_: specific surface area, *V*_tot_: total specific volume of pores, *D*_CO_2__: desorbed amount of CO_2_.

MO	anion of precursors	water amount, dm^3^	*S*_BET_, m^2^/g	*V*_tot_, mm_liq_^3^/g	*D*_CO_2__, μmol/g	ester content (one-step), wt %
MgAl	Cl^–^	0.25	88	394	50.4	22.1
		3	141	511	47.5	20.0
		5	152	487	69.7	19.4
MgFe	Cl^–^	0.25	42	233	45.6	55.4
		3	60	317	41.7	54.2
		5	63	11	45.4	54.0
	NO_3_^–^	0.25	135	544	445.5	49.4
		3	136	624	375.6	48.1
		5	138	626	466.1	44.4

a *S*_BET_: specific surface area, *V*_tot_: total specific volume of pores, *D*_CO_2__: desorbed amount of CO_2_.

The *S*_BET_ was used to determine the specific surface of MOs. The specific surface was slightly increasing for the MgFe MOs synthesized from nitrates (135–138 m^2^/g), independent of the amount of washing water used for synthesis. In contrast, the increasing volume of redistilled water for washing increased both the *S*_BET_ and *S*_meso_ (the specific surface area of mesopores) of MOs synthesized from chlorides (Table 3). The white NaCl ‘flakes’ observed in SEM likely blocked nitrogen (during isotherm analysis) from entering the pores of the MO, leading to a reduced *S*_BET_. With increasing amounts of washing water, NaCl was removed, which reopened the pores and resulted in higher *S*_BET_ values. A similar effect was observed by Titich et al. who synthesized MOs from chloride precursors. Anion-exchanged MOs (Cl^–^ for CO_2_^3–^) exhibited larger pore volumes and increased basicity (determined by TPD-CO_2_ and TPD-SO_2_ measurements).^36^

In comparison, Xu et al. synthesized the MgFeCl mixed oxide with a molar ratio of 4:1 and the specific surface was determined as 75–110 m^2^/g.^37^ Pattanaik et al. studied the properties of mixed oxides from Mg–Ba and they found that the decreasing Ba content resulted in an increase of *S*_meso_ from 18 to 53 m^2^/g.^38^ Fan et al. prepared Zn–Al–In from nitrates and obtained an *S*_meso_ of 59–66 m^2^/g.^39^ Pan et al. observed type IV isotherms in NiCoFe mixed oxides from nitrates with specific surfaces ranging from 67 to 94 m^2^/g.^40^ Wang et al. used the bifunctional oxidic catalyst CaO–SrO synthesized by mixing and gelling in water. They determined a type II isotherm with an *S*_BET_ of 124 m^2^/g for Ca–Sr materials and only 27 m^2^/g for CaO.^41^ Other types of isotherms can be caused by different synthesis methods. Therefore, the specific surface area depends on many parameters and has been reported in a wide range of studies.

The total specific volume of pores was higher for materials synthesized from nitrates (544–626 mm_liq_^3^/g) than for materials synthesized from chlorides (394–511 mm_liq_^3^/g for MgAl MOs and 11–317 mm_liq_^3^/g for MgFe MOs). The volume of micropores was negligible in all materials (0–4 mm_liq_^3^/g).

**Figure 4:**
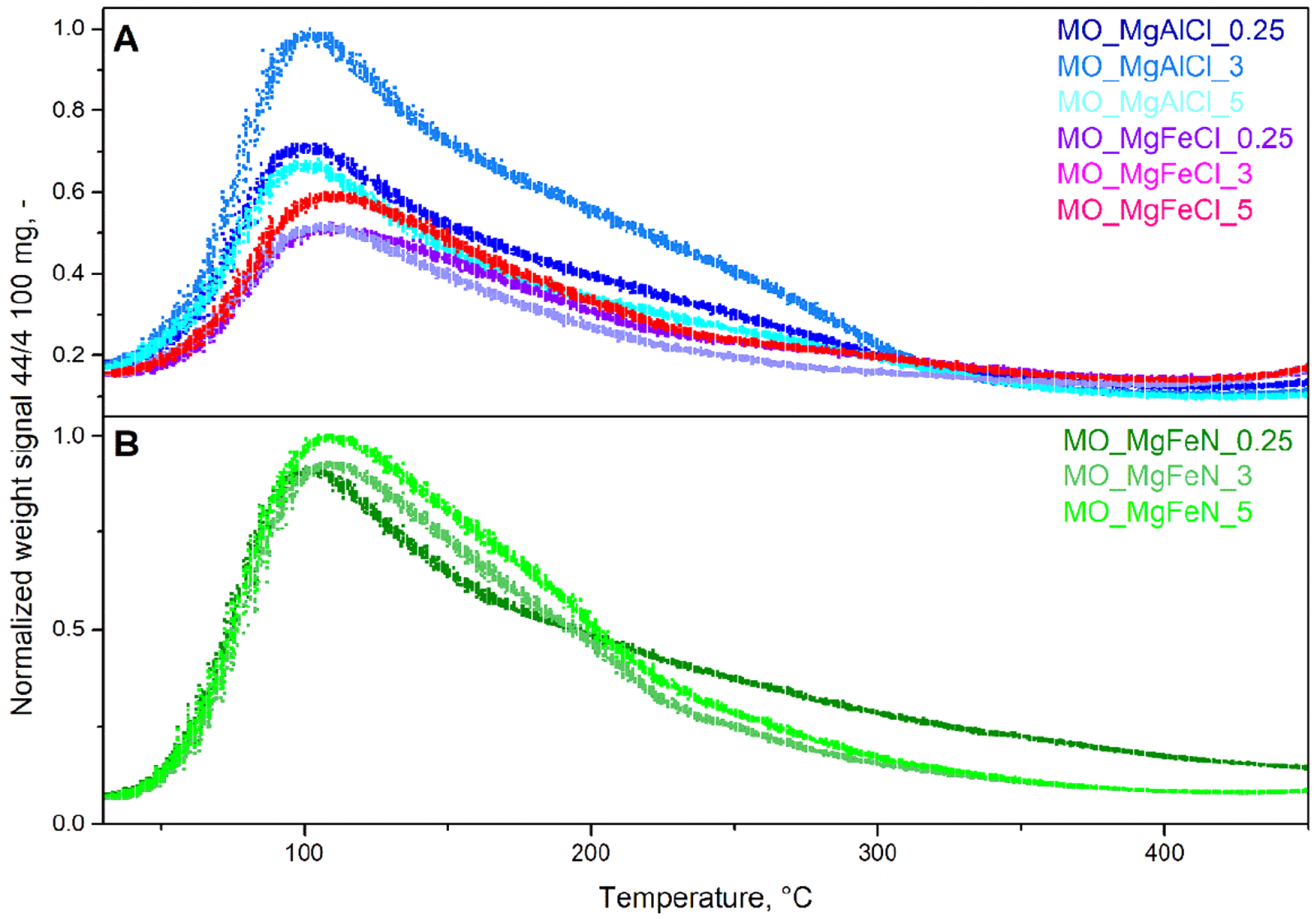
Normalized TPD-CO_2_ profiles of mixed oxides.

The basicity of synthesized MOs was determined by TPD-CO_2_ (Figure 4A,B). The amount of desorbed CO_2_ of mixed oxides was much lower for chloride (for MgAlCl, it was in the range 48–70 μmol/g and for MgFeCl, it was in the range 41–46 μmol/g, respectively) than for nitrate precursors (375–466 μmol/g); see Table 3 (*D*_CO_2__). The lower amount of desorbed CO_2_ can be explained by the presence of NaCl, which (i) remains stable during calcination since 450 °C is insufficient for its decomposition, (ii) lacks basic properties and therefore does not adsorb CO_2_, and (iii) likely blocks the pores, preventing CO_2_ from accessing them. A similar effect was observed by Tichit et al. who found that the substitution of chlorides for carbonates resulted in an increased basicity of MgAl MOs, probably due to chloride anions blocking pores from being accessed by CO_2_.^42^ On the other hand, the formed NaNO_3_ for nitrate precursors is not stable and decomposes to basic Na_2_O, which increases the basicity of the catalyst (previously described in the study^18^).

In comparison, Chaillot et al. performed the TPD-CO_2_ experiment on MgAl mixed oxides from chloride and acetylacetonate precursors with molar ratios of 3:1 (Mg/Al). The measured basicity of their MOs was much higher (from 386 to 527 μmol/g) probably because their hydrotalcites were not washed with distilled water but only centrifuged to separate the MOs from the NaOH solution (therefore, NaOH probably remained in HTs). However, no analysis in detail was carried out; only the microcalorimetry exhibited predominantly strong basic sites.^26^ Gao et al. studied the CO_2_ desorption process in MgAlCl mixed oxides with various molar ratios of metals (from 1.0 to 4.5). They achieved a desorption maximum of CO_2_ in materials with molar ratios of 3.0:1 (Mg/Al).^43^ Similarly, predominantly strong basic sites were observed by TPD-CO_2_ measurement with TGA-DSC analysis in Mg–Zn–Zr MO synthesized by Tichit et al.^44^ The basicity increased with increasing Mg and Zr^4+^ content. In a different study, Podila et al. synthesized MgFe MOs from nitrates with a molar ratio of 3:1 for Mg/Fe. They found that their materials exhibited quite high basicity (994 μmol/g) but their measurement was carried out up to 600 °C, whereas the calcination was only carried out to 450 °C. So, the measured ‘basicity’ was influenced by higher temperatures (>450 °C) because various compounds might decompose in the heating process. Therefore, the whole signal cannot be attributed to basic sites/properties.^45^ A similar research was carried out by Xu et al. who synthesized MgFe mixed oxides from nitrates with molar ratios from 2:1 to 5:1 (Mg/Fe). The amount of desorbed CO_2_ was in the range of 460–570 μmol/g.^46^

It is generally accepted that weak, medium, and strong basic sites are present in mixed oxides. However, the distinction between them is not clear and depends on the paper. Nicoto F. attributes TPD-CO_2_ signals (i) between 17 and 157 °C to weak basic sites, (ii) between 158 and 397 °C to medium-strength sites, and (iii) above 397 °C to strong basic sites.^47^ Goda attributes the signals as (i) weak basic sites (<150 °C), (ii) intermediate basic sites (150–300 °C), and (iii) strong basic sites (>300 °C).^48^ Aldureid attributed the strength of sites as (i) weak (temperature of desorption below 200 °C), (ii) intermediate (temperature of desorption in the range 201–499 °C), and (iii) strong (temperature of desorption above 500 °C).^1^ These discrepancies make a comparison of various papers difficult. All MOs synthesized in this paper have a similar occurrence of weak and strong basic sites without a clear distinction between them.

### Catalytic Activity in Transesterification

First, the one-step transesterification was carried out (120 °C, 6 h), and the ester content was determined (Table 3). The acid number of oil was very low (0.15 mg KOH/g) and so formation of soaps was negligible. Note that due to the relative low yield of methyl ester, the glycerol phase was not formed. The lowest ester content (19.4–23.3 wt %) was obtained with MO_MgAlCl catalysts, which can be attributed to their relatively low basicity (47–70 μmol/g of CO_2_ desorbed) and the predominance of small pores (8–20 nm in diameter) because the relatively large molecules of triacylglycerols do not enter the pores. The small pores were likely caused by a high amount of sodium in the form of NaCl (confirmed by XRD and EDX), which caused pore blocking. For MgFe MOs, the ester content for chloride precursors is higher (54.0–55.4 wt %) than for nitrate precursors (44.4–49.4 wt %), although the basicity of MgFeCl is much lower (≈45 mmol/g compared to ≈400 mmol/g for nitrates; Table 3). The ester content depends much less on the amount of washing water used for chlorides than for nitrate precursors because the formed NaCl is stable through calcination and does not influence the transesterification (it has no catalytic effect). For nitrates, the formed NaNO_3_ (during the synthesis) is transformed to Na_2_O in calcination and then to NaOH (in reaction with water). NaOH then acts in transesterification as a homogeneous catalyst, increasing the ester content (confirmation by sodium decreasing, Table 2) and a less formed homogeneous catalyst and so less ester content. In comparison, the yield for MgAl mixed oxides (from nitrate precursors) without almost any residual sodium impurities (thoroughly washed) was only 19 wt %, as published in the previous paper.^16^ This confirmed that without the presence of NaNO_3_, the yield is very low. No change in the structure of materials after transesterification was observed. The metal content in the ester phase was determined for all of the less washed MO (Table S3 in the Supporting Information). Fe and Al were negligible (4 mg/kg), but the contents of Na and Mg were much higher because they are more easily soluble.

Moreover, the two-step transesterification was carried out for the catalysts with the highest ester content in the previous (one-step) transesterification (MO_MgFeCl). After the first step, the yield of methyl esters was slightly higher than for the one-step reaction (due to the higher reaction temperature): 58.1, 62.6, and 57.4 wt % for MO_MgFeCl_0.25, MO_MgFeCl_3, and MO_MgFeCl_5, respectively. It further increased to 84.7, 84.1, and 80.8 wt %, respectively, after the second transesterification step. The metal content was determined for the less washed MO (MgFeCl_0.25) in the ester (Table S3 in the Supporting Information) and also in the glycerol phases (formed in a small amount). In the ester phase, Na and Mg were higher than only for the one-step transesterification, probably due to higher temperatures (Fe is negligible, 4 mg/kg). Moreover, the content of Na and Mg (in both phases) is higher after the first step than after the second step because more easily accessible metals leached first. In the glycerol phase, the content of Na and Mg is much higher because its higher polarity increases the solubility of the polar ions: Na (21.4 mg/g) and Mg (2.5 mg/g) after the first step and Na (19.6 mg/g) and Mg (0.8 mg/g) after the second step.

The comparison with other papers is quite difficult because the conditions of catalyst synthesis and the transesterification reaction are very different. Transesterification is carried out at temperatures from 60 to 200 °C, molar ratios of methanol to oil from 2:1 to 40:1, pressure from atmospheric to 4 MPa, time from 2 to 6 h, and a catalyst amount of 2–6 wt %. The type of reactor (batch or flow) is different as well as the transesterification process for the batch reactor (one stage or two stages).^49,50^ The type of heating (conventional or microwave-aided) can also be used.^51^ Moreover, catalyst activity is determined by various ways, such as by the ester yield, by the content of esters, or by oil conversion. Kutalek et al. published the ester yield for the Mg–Al catalyst from 55 to 60% at the fixed-bed reactor at 115 °C and 4 MPa 76% at 140 °C after 50 h.^52^ Salinas et al. used La_2_O_3_–Al_2_O_3_ MOs at an atmospheric pressure and at 65 °C, and after 5 h, it reached a yield of 45%.^53^ Xu et al. synthesized MgFe MOs by the urea method; the transesterification was carried out with methanol and microalgae oil (molar ratios from 2:1 to 6:1) at 60 °C for 90 min. They obtained the yield of 88%.^46^ Duangdee et al. obtained a high conversion of 99.6%, but at harsh reaction conditions (200 °C, 3.9 MPa, molar ratio of methanol to palm oil: 10:1, and 3 h) with oxides of metals Ce, Nd, Y, and La.^54^ Other authors used MoZnFe MOs for transesterification of waste frying oil with a FAME high yield 97.6%, but at 180 °C, a high molar ratio of methanol to oil (40:1), 6 wt %, and 3 h.^55^

## Conclusions

The influence of sodium impurities on the properties of mixed oxides (MgAl and MgFe), which remained after the synthesis from hydrotalcites, was studied. The hydrotalcites were synthesized from two types of precursors (nitrates and chlorides), and various amounts of water were used for washing, which influenced the content of sodium impurities. All synthesized materials were characterized using many methods. It was found that hydrotalcites synthesized from nitrates contained NaNO_3_, which was decomposed to Na_2_O (through calcination) and rapidly increased the basic properties of catalysts and so the yield of transesterification. On the contrary, the formed NaCl from chloride precursors was stable and inactive in the reaction. It also decreased the, average pore size, and basicity, resulting in a decreased yield of methyl esters. Therefore, the removal of impurities from materials should be emphasized using sufficient amounts of washing water. The sodium content should be determined in mixed oxides to determine successful washing.

## Supplementary Material



## Notes

Chemical composition on the surface of mixed oxides, EDX profiles of mixed oxides, chemical composition of ester phases, and sphericity diagrams (PDF)

## Abbreviations

HTs: hydrotalcites (layered double hydroxides)
MOs: mixed oxides
MEs: methyl esters
XRD: X-ray diffraction
GC-FID: gas chromatography with a flame ionization detector
SEM-EDX: scanning electron microscopy with energy-dispersive X-ray spectroscopy
TPD-CO_2_: temperature-programmed desorption with CO_2_
ICP-MS: inductively coupled plasma with mass spectrometry
*S*_BET_: specific surface area (m^2^/g)
*S*_meso_: specific surface area of mesopores (m^2^/g)
*V*_tot_: total specific volume of pores (mm_liq_^3^/g)
*D*_CO_2__: desorbed amount of CO_2_ (μmol/g)
