# Supplementary material for: Mixed Oxides: Role of Washing and Residual Ions in Transesterification Reactions
Source: ACS Omega. 2025 Oct 28;10(44):53341–9. doi: 10.1021/acsomega.5c08243 (PMC12612951; doi:10.1021/acsomega.5c08243)

Supporting Information

Mixed Oxides: Role of Washing and Residual Ions in Transesterification Reactions

David Kocián[1], Martin Hájek[1,], Karel Soukup[2], Luděk Kaluža[2], Rostislav Prokeš[3], Miroslava Bérešová[4], Jakub Vagunda[1]*

[1] University of Pardubice, Faculty of Chemical Technology, Studentská 573, 532 10 Pardubice, Czech Republic

[2] Institute of Chemical Process Fundamentals of the CAS, Rozvojová 135, 165 00 Prague 6, Czech Republic

[3] VSB – Technical University of Ostrava, Centre for Energy and Environmental Technologies, ENET Centre 17. listopadu 2172/15, 708 00 Ostrava, Czech Republic

[4] Slovak University of Technology, Faculty of Chemical and Food Technology, Radlinského 9, 812 37 Bratislava, Slovak Republic

[*] corresponding author: martin.hajek@upce.cz

Table of contents:

Table S1. The chemical composition on the surface of mixed oxides (determined by EDX)......2
Table S2. The EDX profiles of mixed oxides......3
Table S3. The chemical composition of ester phases (determined by ICP-MS)......4
Figure S1. The sphericity diagram of MgAlCl (A), MgFeN (B) and MgFeCl (C) for MO......5

Table S1. The chemical composition on the surface of mixed oxides (determined by EDX)

Mixed oxide	Mg, wt.%	Al, wt.%	Fe, wt.%	Na, wt.%	Cl, wt.%	N, wt.%	O, wt.%
MO_MgAlCl_0.25	32.0	5.6	-	2.4	7.1	-	41.6
MO_MgAlCl_3	27.1	8.2	-	0.4	4.8	-	44.4
MO_MgAlCl_5	30.2	9.6	-	0.1	4.8	-	44.2
MO_MgFeCl_0.25	19.0	-	17.8	5.3	10.4	-	30.9
MO_MgFeCl_3	25.0	-	23.8	-	2.2	-	38.3
MO_MgFeCl_5	27.2	-	26.7	-	2.5	-	34.6
MO_MgFeN_0.25	27.8	-	18.9	0.6	-	0.7	40.4
MO_MgFeN_3	25.0	-	25.7	-	-	0.1	38.8
MO_MgFeN_5	27.9	-	24.9	0.1	-	0.2	37.0

Table S2. The EDX profiles of mixed oxides

HT	Precursors	Washing water, dm^3	Mg	Al	Fe	Cl	N	Na
		0.25	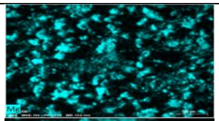	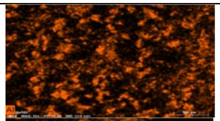	-	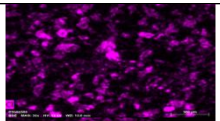	-	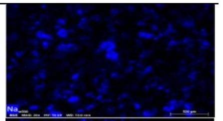
MgAl	Cl	3	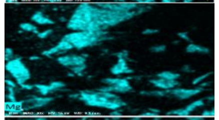	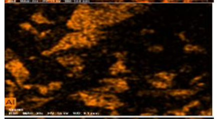	-	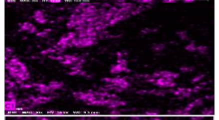	-	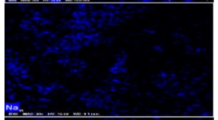
		5	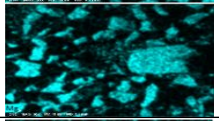	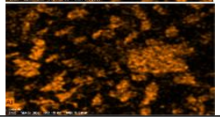	-	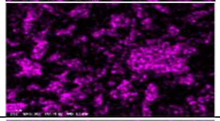	-	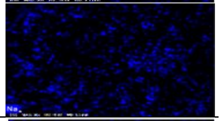
MgFe		0.25	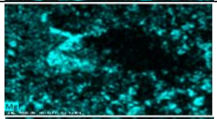	-	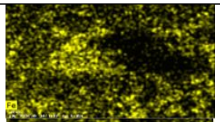	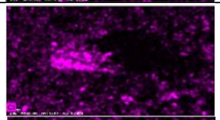	-	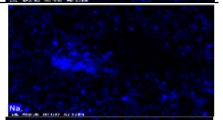
	Cl	3	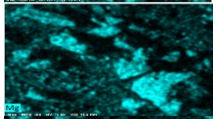	-	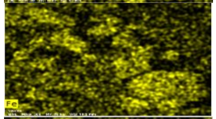	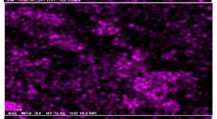	-	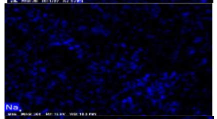
		5	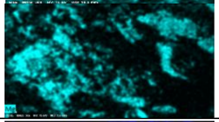	-	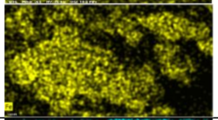	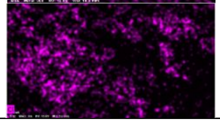	-	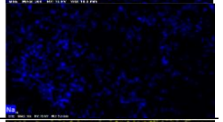
		0.25	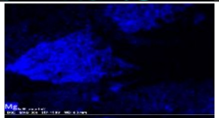	-	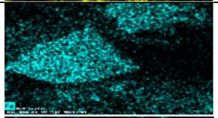	-	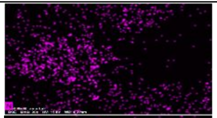	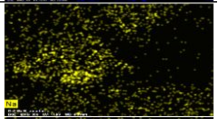
	NO_3	3	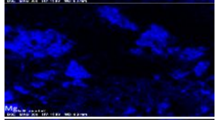	-	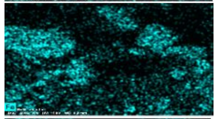	-	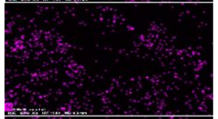	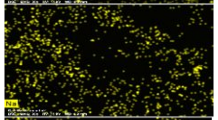
		5	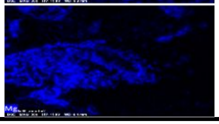	-	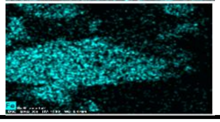	-	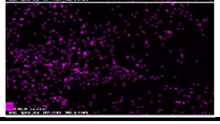	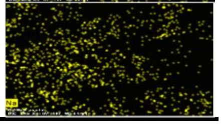

Table S3. The chemical composition of ester phases (determined by ICP-MS)

Mixed oxide	Mg, mg/kg	Al, mg/kg	Fe, mg/kg	Na, mg/kg
MO_MgAlCl_0.25	43	4	-	47
MO_MgFeCl_0.25	28	-	4	50
MO_MgFeN_0.25	19	-	4	53
MO_MgFeCl_0.25 (first step)	62	-	5	68
MO_MgFeCl_0.25 (second step)	34	-	4	31

Figure S1. The sphericity diagram of MgAlCl (A), MgFeN (B) and MgFeCl (C) for MO

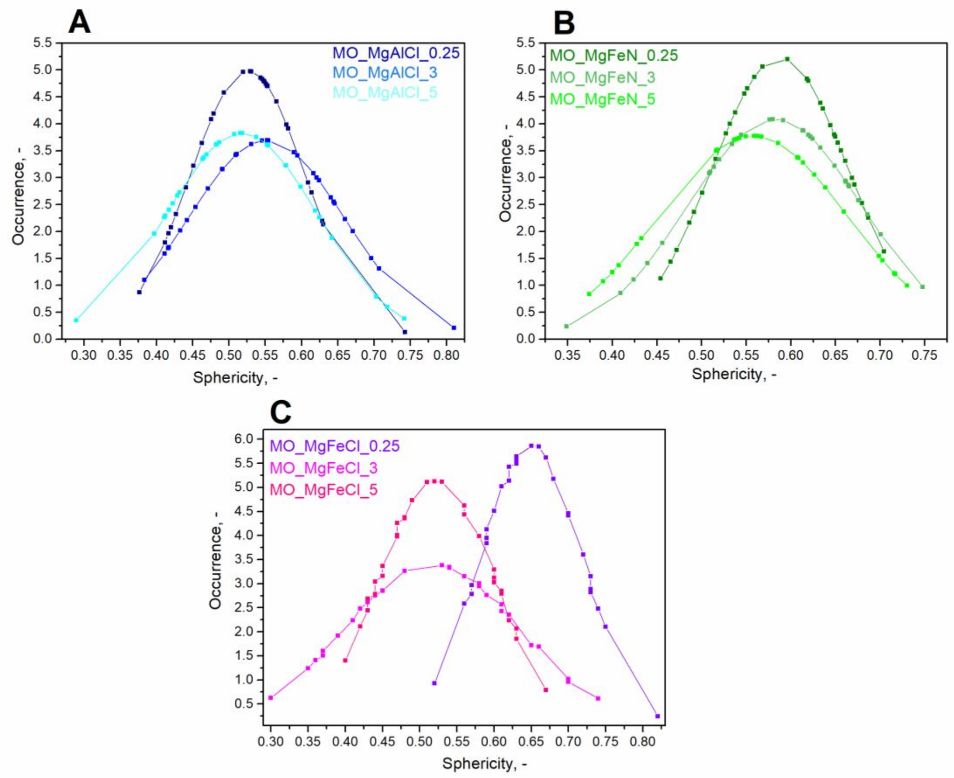

Supplement: Supplementary file 1 [file ao5c08243_si_001.pdf]
